# Thyroid Cancer Survival in the Multiethnic Cohort Study

**DOI:** 10.3390/ijerph21030324

**Published:** 2024-03-10

**Authors:** Janine V. Abe, Song-Yi Park, Christopher A. Haiman, Iona Cheng, Loïc Le Marchand, Brenda Y. Hernandez, Lynne R. Wilkens

**Affiliations:** 1 Cancer Epidemiology Program, University of Hawaiʻi Cancer Center, Honolulu, HI 96813, USA; spark@cc.hawaii.edu (S.-Y.P.); loic@cc.hawaii.edu (L.L.M.); brenda@cc.hawaii.edu (B.Y.H.); lynne@cc.hawaii.edu (L.R.W.); 2 Department of Preventive Medicine, Keck School of Medicine, University of Southern California, Los Angeles, CA 90033, USA; christopher.haiman@med.usc.edu; 3 Department of Epidemiology and Biostatistics, University of California San Francisco, San Francisco, CA 94158, USA; iona.cheng@ucsf.edu

**Keywords:** disparities, race, ethnicity, survival, thyroid cancer

## Abstract

Objective: The US 5-year survival rate after thyroid cancer (TC) diagnosis is over 95%. Our aim was to investigate survival differences by sex and race and ethnicity in a multiethnic US population. Design: In the Multiethnic Cohort (MEC) study, a total of 605 incident TC cases were identified by linkage to HI and CA statewide cancer registries. Cox models were performed to compare the risk of all-cause mortality among TC cases by sex and race and ethnicity, with adjustment for age, first course of treatment, baseline body mass index, smoking status, alcohol intake, and neighborhood socioeconomic status. Survival among cases was also compared to matched MEC controls with no thyroid cancer. Results: After a mean follow-up of 10.1 years, 250 deaths occurred among TC cases, including 63 deaths attributed to thyroid cancer. The median survival was 14.7 years, and the 5-year age-adjusted overall survival was 84.4% for female cases and 68.7% for male cases (*p* < 0.0001, HR 2.28 (95% CI: 1.72, 3.01)). Age-adjusted survival was lower among African American, Native Hawaiian, and Filipino cases, compared to Japanese American cases, with Whites and Latinos being intermediate. Men and Filipinos were found to have excess mortality due to thyroid cancer compared to controls (adjusted HR 1.39, 95% CI: 1.11, 1.74; HR 1.62, 95% CI: 1.04, 2.53, respectively). Conclusions: Sex and racial and ethnic disparities in survival among TC cases were similar to those found in the general population. However, cases with TC had an excess risk of death among males and for Filipinos.

## 1. Introduction

Thyroid cancer (TC) is the most common endocrine cancer worldwide, and incidence rates have been increasing in most countries [1]. This cancer is ranked as the 9th most common in the world [2], and 12th in the United States (US) [3]. Sex and racial and ethnic disparities are observed with TC. Women have higher incidence rates than men, with a ratio of 3:1 [4]. In US SEER data, Whites (15.1 per 100,000) [4] and Filipinos (19.6 per 100,000) [5] have higher incidence rates than other race and ethnic groups (8.4 African American and 12.2 Hispanic per 100,000), and, in Hawaiʻi SEER data, the rates for Filipinos are also elevated [6]. The estimated number of deaths due to TC in the US in 2023 is 2120 (970 men; 1150 women) [7]. The overall US 5-year survival rate for TC is 98.3% [3]. Sex and racial and ethnic disparities have been reported, with lower survival in men than in women [8] and Non-Hispanic Blacks (NHB) compared to Non-Hispanic Whites (NHW) [8]. However, there is limited literature on survival in TC cases among other racial and ethnic groups. This manuscript utilizes the Multiethnic Cohort (MEC) to compare TC survival across sex and racial and ethnic groups, adjusted for relevant risk factors.

## 2. Materials and Methods

### 2.1. Study Population

The MEC is a prospective cohort designed to identify lifestyle and genetic risk factors for cancer in a diverse population, as has been previously described [9]. In short, between 1993 and 1996, a baseline questionnaire (QX1) was mailed to adults aged 45–75 residing in Hawaiʻi and California (mostly Los Angeles County). The baseline questionnaire surveyed demographic characteristics, diet through a food frequency questionnaire, anthropometrics, smoking history, reproductive history, and other lifestyle factors. The MEC was designed to study the major racial and ethnic groups in the U.S. including African American, Japanese American, Latino, Native Hawaiian, and White. Filipinos were disaggregated from the Other racial and ethnic group for this report because of their high incidence of TC [5,6]. Race and ethnicity was defined by self-report. Native Hawaiian included any-part Hawaiian, as is traditional in this native group; 94% of the other MEC participants reported a single race and ethnicity. The remaining participants were assigned either to one of the six groups of interest if it was included in their admixture or to the “Other” group. This study was approved by the Institutional Review Boards of the University of Hawaiʻi and the University of Southern California.

### 2.2. Thyroid Cancer Ascertainment

TC cases in the MEC were identified by linkage to the statewide Surveillance, Epidemiology, and End results (SEER) statewide cancer registries of Hawaiʻi and California [9]. This passive approach should identify virtually all cases, as migration outside the two states has been low. Included were cases with primary invasive thyroid cancers with an ICD-O3 code of C730-C739 diagnosed after cohort entry. The SEER Summary Stage 2018 [10] was used, categorized as localized, regional, or distant. Information on the first course of treatment consisted of thyroid hormone, radiation, and surgery. Deaths were identified via linkages with the National Death Index and death certificate files for Hawaiʻi and California [9], in addition to the cancer registries. Cancer and death ascertainment were complete through 31 December 2017.

### 2.3. Statistical Analysis

Five-year and ten-year survival rates were estimated overall and by sex and race and ethnicity. Survival time was calculated as years starting at the date of the TC diagnosis and ending at either (1) date of TC-specific death, (2) date of death due to other causes, or (3) the closure date for death reporting (31 December 2017). Participants who were alive at the closure date were censored at that time. The analysis of TC-specific death is not presented as the number (*n* = 63) was too small, especially in subgroups.

Survival estimates by sex and by racial and ethnic groups were computed from Cox proportional hazards models, with age as the time metric. Minimal adjustment included age at diagnosis as a covariate, as well as sex and race and ethnicity where appropriate. Intermediate adjustment additionally included stage (localized, regional, distant) as a strata variable, and full adjustment additionally included the first course of treatment (thyroid hormone, radiation, surgery), and baseline values from QX1 for body mass index (BMI) (≤25, 25–29.9, ≥30 kg/m^2^, based on self-reported height and weight), smoking status (former, never, current), alcohol intake (g/day, continuous), and neighborhood socioeconomic status (nSES). At baseline, nSES was created via addresses that were geocoded and linked to the 1990 US Census block groups. The nSES score was previously developed from principal component analysis which included 7 census indicator variables: poverty, education, household income, rent, house value, employment, and occupation [11]; nSES were parameterized as low (Quintile 1 to 3) and high (Quintile 4 and 5) based on Hawaiʻi and Los Angeles County specific distributions [12]. These adjustment factors have been found to have an association with survival in the literature or in prior MEC studies. Race and ethnicity were categorized as African American (AA), Filipino, Japanese American (JA), Latino, Native Hawaiian (NH), White, and Other (e.g., Chinese and Korean). Japanese American cases were designated as the reference group as they comprised the largest group with the lowest risk of death. The proportional hazards assumption, tested based on the interaction of time and the exposure, was met for all variables but stage, which was included as a strata variable. Hazard ratios (HR) and corresponding 95% confidence intervals (CI) are reported. Due to small numbers, men and women were combined to study racial and ethnic differences, adjusting for sex in the model. The Other race category is not presented separately due to small numbers (12 cases); however, the group is included in the total. As a sensitivity analysis, the comparisons by race and ethnicity were performed for men and women separately. Given the small number of TC-specific deaths, to study whether TC cases experienced excess mortality, we compared the overall survival of TC cases with MEC members with no TC within sex and racial and ethnic groups. Each case was randomly matched to up to 100 controls who were alive at the age and date of their matched case’s diagnosis; the matching factors were sex, race and ethnicity, and birth year (±5 years). Survival time for this analysis was calculated as years starting at the date of the case’s diagnosis within the matched set and ending at the date of death or closure date of 31 December 2017. A Cox model of the risk of dying was fit to estimate the HR for TC cases versus controls, with matched set as a strata variable and adjustment for the baseline values for BMI, alcohol use, smoking status, and nSES. Other covariates, such as pack-years, were considered but found not to improve the model fit. Accounting for the clustering of nSES by the census block group did not alter the results. Significance was defined as *p* < 0.05, and all analyses were conducted using SAS version 9.4 (SAS Institute, Cary, NC, USA).

## 3. Results

A total of 605 primary incident invasive TC cases were included in the analysis, with the majority, 82.5%, having papillary and mixed papillary follicular tumors. During a mean follow-up of 10.1 years, a total of 250 deaths were identified, with 63 deaths due to TC. The characteristics of the cases by race and ethnicity are displayed in Table 1. The majority (70%) of cases were women. The mean age of diagnosis ranged from 66.5 years in NH to 72.0 years in AA. Obesity, smoking status, nSES, and alcohol intake differed across racial and ethnic groups. The majority of TC cases were diagnosed at a localized stage, except among Filipinos that had more regional and distant tumors. The vast majority of cases received surgery as the first course of treatment (89%); about half of cases had radiation therapy, which was most common among Filipinos and JA cases.

**Figure 1:**
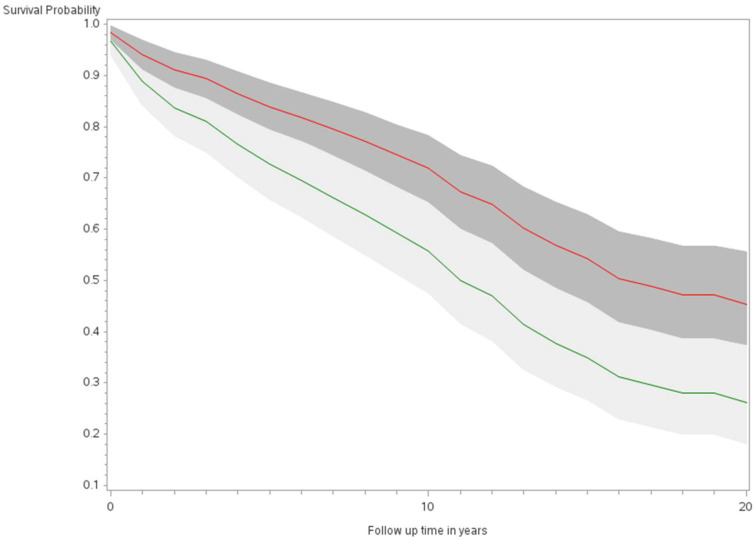
Minimally adjusted survival curves for all-cause mortality among thyroid cancer cases by sex in the Multiethnic Cohort study 1993–2017. (*p* < 0.0001) Green curve = male (1); Red curve = female (2).

**Figure 2:**
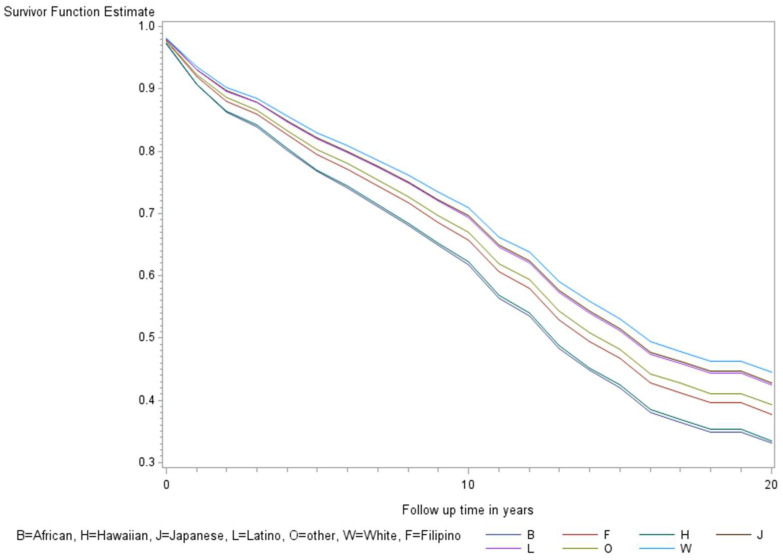
Minimally adjusted survival curves for all-cause mortality among thyroid cancer cases by race and ethnicity in the Multiethnic Cohort study 1993–2017. (*p* = 0.008) B = African American, F = Filipino, H = Native Hawaiian, J = Japanese American, L = Latino, O = Other, W = White.

**Figure 3:**
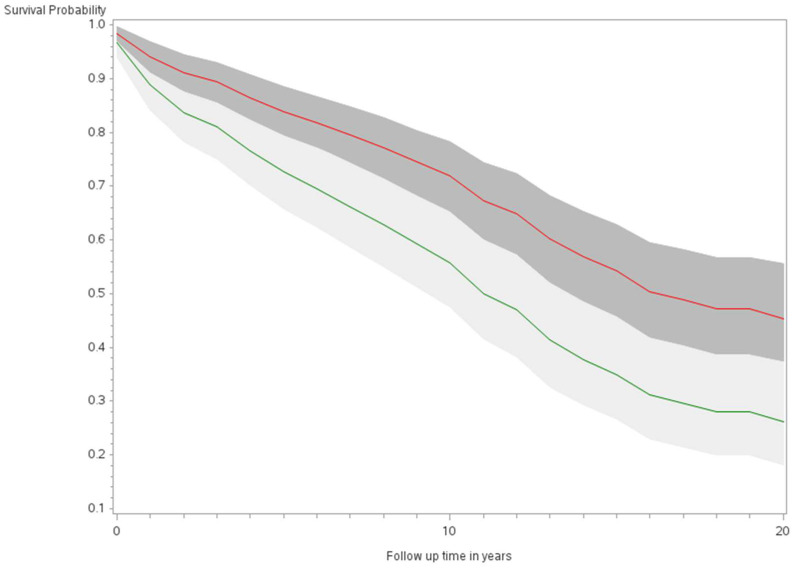
Fully adjusted survival curves for all-cause mortality among thyroid cancer cases by sex in the Multiethnic Cohort study 1993–2017. (*p* < 0.001) Green curve = male (1); Red curve = female (2).

**Figure 4:**
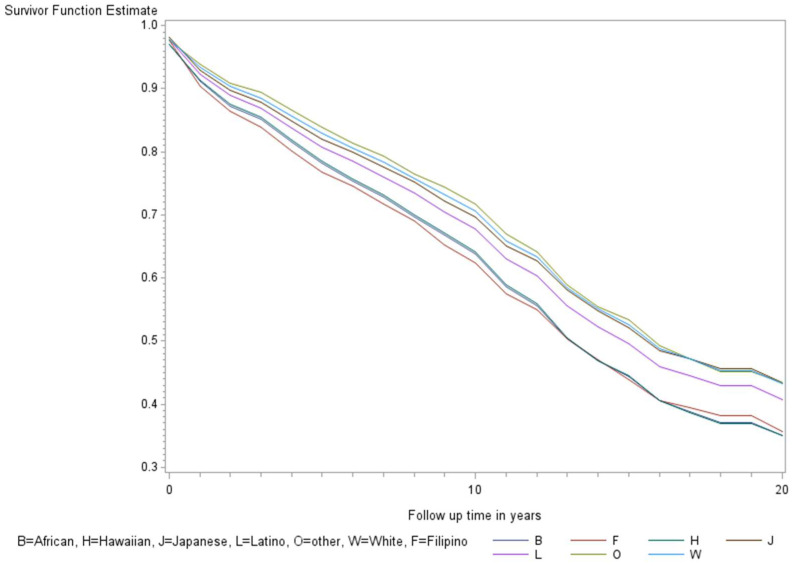
Fully adjusted survival curves for all-cause mortality among thyroid cancer cases by race and ethnicity in the Multiethnic Cohort study 1993–2017. Adjusted for the age of diagnosis, race and ethnicity, sex, BMI, alcohol, smoking, therapy, stage, and nSES. (*p* < 0.001) B = African American, F = Filipino, H = Native Hawaiian, J = Japanese American, L = Latino, O = Other, W = White.

The age-adjusted survival curves from Cox regression among TC cases are shown by sex in Figure 1 and by race and ethnicity in Figure 2. Male cases had a significantly higher risk of death than female cases (*p* < 0.0001) with an HR of 2.28 (95% CI: 1.72, 3.01) (Table 2). The 5-year minimally adjusted survival was 84.4% for women and 68.7% for men; the 10-year survival estimates were 73.2% and 52.0%, respectively (Table 2). The risk of death was observed to be significantly higher in each of the racial and ethnic groups compared to JA: at least 80% higher for AA, NH, and Filipino cases (Table 2). The fully-adjusted survival curves for all causes are shown by sex in Figure 3 and by race and ethnicity in Figure 4; the survival estimates were similar to the age-adjusted values. The elevated risk remained after multivariable adjustments for AA, NH, and Filipino cases, although statistical significance was maintained only in NH cases (Table 2). No single covariate produced attenuated results. When cases with localized and regional/distant stages were analyzed separately, the relative risk for men was significantly higher than women for all stages, while each racial and ethnic group had a significantly higher risk than JA cases in the regional/distant stage only (Supplemental Table S1).

Comparing TC cases’ risk of dying to that of matched controls, there was little difference in the risk of death overall (*p* = 0.94) (Table 3). Survival for female TC cancer cases was similar to the matched controls; however, among men, a TC diagnosis led to a 39% increased risk of death (adjusted HR = 1.39, 95% CI: 1.11, 1.74). Survival for TC cases was similar to matched controls for all racial and ethnic groups, except for Filipino adults, where TC cases had a statistically significant increased risk of death (adjusted HR = 1.62, 95% CI: 1.04, 2.53).

## 4. Discussion

In our analysis of TC cases, we found sex and racial and ethnic differences in survival, with a significantly higher risk of death in men compared to women and in each of the other racial and ethnic groups compared to JA cases. Additionally, an excess risk of death was found among male and Filipino TC cases compared to age-, sex-, and race and ethnicity-matched controls with no TC diagnosis. Similar sex differences were observed in a study using the National Thyroid Cancer Treatment Cooperative Study Group Registry that observed higher survival in women than men, for all (88% vs. 71%) as well as disease-specific (96% vs. 91%) causes [13]. Our results for racial and ethnic group differences are in agreement with a report using the US SEER 1994–2004 for the papillary subtype, where survival was the lowest in Blacks (91.5%), followed by Asian Pacific Islanders (API, 94.4%) and NHW (95.3%) [14]. A similar lower survival was observed for Filipinos, using the California Cancer Registry; the 5-year overall survival for Filipinos was 95.1% compared to non-Filipino Asians at 97.1% and non-Asians at 96.3% [15]. Similarly, Hawaiʻi statewide mortality rates for TC were higher in men compared to women [16]. Using the US SEER data for 1998–2005, the relative risk (RR) for death among TC cases with the regional stage in men compared to women was 1.56 (95% CI: 1.31, 1.85) [17], and for 2004–2018, the RR for males versus females was 1.47 (95% CI: 1.25, 1.74) for TC-specific death and 1.63 (95% CI: 1.50, 1.78) for all-cause death [18].

The difference in TC survival by sex has been suggested to be due to ascertainment bias, with men seeking healthcare services at an older age and later stage of tumor and women utilizing care more frequently [13]. In the MEC, men were diagnosed at an older age (71 vs. 69 years) and had more regional and distant disease compared to women (36.5% vs. 27.3% regional; 13.2% vs. 8.7% distant). Also, an earlier report of the incidence of TC in Hawaiʻi noted that women were diagnosed at a younger age than men, and men were diagnosed more often at a more advanced stage [19]. Mortality rates in the US population, in general, show that men have 40% higher death rates compared to women, which has been related to unhealthy behaviors and irregular checkups [20]. However, we found that men experienced excess death after a TC diagnosis, compared to men with no TC, matched on race and ethnicity and age. In general, NHB have higher mortality rates compared to NHW [21,22] and AAPI [23]. In an earlier report of Cancer at a Glance in Hawaiʻi, NH and Filipinos in Hawaiʻi had higher overall TC mortality rates compared to other races and ethnicities and other Asian subgroups (JA/Chinese) [24]. Racial and ethnic differences for mortality have been associated with social determinants of health [23].

The disparity in survival among TC cases was compared with those in the general MEC control population. Men with a diagnosis of TC were found to have higher death rates than similar men with no diagnosis. In addition, the overall disparities in survival among TC cases by race and ethnicity largely mirror the survival in the general population among African Americans and Native Hawaiians. The general excess mortality among these racial and ethnic minorities has been related to SES, screening, preventive measures, and treatments, as well as health-related behaviors (smoking, drinking, diet) [25]. Filipinos with TC were found to exhibit excess mortality compared to Filipinos who did not have the disease. The excess risk observed for men and Filipinos may be partially related to their later stages at diagnosis.

The limitations of our study to understand the extent of disparities in TC survival include the limited number of deaths due to TC. In addition, we lack detailed information on treatment, including adherence or the use of native medicines, and on tumor markers. The strength of our study includes a diverse disaggregated multiethnic population, including high-risk groups, with information on the age at diagnosis, stage and histology, and many risk factors for death. The identification of cancer cases and deaths through passive linkage to statewide registries in Hawaiʻi and California is likely to be virtually complete in the MEC populations which had little out-migration to other states. Our results suggest that the survival disparities among racial and ethnic groups in TC cases mirror those in the general population for African Americans and Native Hawaiians, but that TC leads to excess death in men and Filipinos. Future studies are warranted to further explore reasons for the poorer survival in men and Filipinos, including the assessment of tumor molecular profiles and germline genetics of cases.

## Figures and Tables

**Table 1:** Characteristics of thyroid cancer cases overall and by race and ethnicity in the Multiethnic Cohort study 1993–2017 ^a^.

	Total	African American	Filipino	Native Hawaiian	Japanese American	Latino	White	*p* ^b^
Cases, n (%)	605	73	54	68	136	156	106	
Men	168 (27.8)	12 (16.4)	17 (31.5)	24 (35.3)	38 (27.9)	32 (20.5)	45 (42.5)	
Women	437 (72.2)	61 (83.6)	37 (68.5)	44 (64.7)	98 (72.1)	124 (79.5)	61 (57.6)	
Deaths, n (%)								
All causes	250	41 (56.2)	24 (44.4)	32 (47.1)	49 (36.0)	61 (39.1)	38 (35.9)	
Thyroid cancer	63	11 (15.1)	4 (7.4)	5 (7.4)	14 (10.3)	22 (14.1)	7 (6.6)	
Age of diagnosis, mean (SD)	69.5 (9.3)	72.0 (9.4)	68.7 (8.6)	66.5 (9.2)	69.3 (10.3)	70.2 (8.9)	68.8 (8.4)	0.01
Years from diagnosis to death, mean (SD)	22.1 (5.9)	22.1 (6.1)	20.7 (7.0)	21.3 (6.2)	22.7 (5.5)	22.2 (6.0)	22.5 (5.3)	0.40
BMI, n (%)								<0.001
<25 kg/m^2^	234 (38.7)	13 (17.8)	31 (57.4)	21 (30.9)	88 (64.7)	34 (21.8)	41 (38.7)	
25–29.9 kg/m^2^	244 (40.3)	38 (52.1)	16 (29.6)	18 (26.5)	37 (27.2)	86 (55.1)	45 (42.5)	
≥30 kg/m^2^	125 (20.7)	21 (28.8)	7 (13.0)	29 (42.7)	11 (8.1)	35 (22.4)	20 (18.9)	
Missing	2 (0.3)	1 (1.4)	-	-	-	1 (0.6)	-	
Smoking status, n (%)								<0.001
Never	318 (52.6)	30 (41.1)	35 (64.8)	26 (38.2)	79 (58.1)	94 (60.3)	46 (43.8)	
Former	205 (33.9)	28 (38.6)	11 (20.4)	22 (32.4)	45 (33.1)	50 (32.1)	47 (44.8)	
Current	72 (11.9)	14 (19.2)	6 (11.1)	19 (27.9)	11 (8.1)	8 (5.1)	12 (11.4)	
Missing	10 (1.7)	1 (1.4)	2 (3.7)	1 (1.5)	1 (0.7)	4 (2.6)	1 (0.9)	
Alcohol, g/day, mean (SD)	4.2 (15.3)	2.4 (6.8)	2.6 (7.3)	5.7 (17.7)	4.1 (18.3)	2.5 (6.5)	7.3 (22.5)	0.16
nSES, n (%)								<0.001
Low (1–3)	307 (50.7)	53 (72.6)	46 (85.2)	45 (66.2)	57 (41.9)	120 (76.9)	44 (41.5)	
High (4–5)	230 (38.0)	20 (27.4)	8 (14.8)	23 (33.8)	79 (58.1)	36 (23.1)	62 (58.5)	
Stage, n (%)								0.01
Localized	347 (57.8)	46 (63.9)	23 (42.6)	45 (66.2)	71 (52.2)	86 (55.5)	67 (63.2)	
Regional	180 (29.9)	11 (15.3)	20 (37.0)	16 (23.5)	56 (41.2)	45 (29.0)	29 (27.4)	
Distant	60 (10.0)	11 (15.3)	8 (14.8)	5 (7.4)	8 (5.9)	21 (13.6)	7 (6.6)	
Unknown	16 (2.7)	4 (5.6)	3 (5.6)	2 (2.9)	1 (0.7)	3 (1.9)	3 (2.8)	
Missing	2 (0.3)	1 (1.4)	-	-	-	1 (0.6)		
Tumor size, n (%)								
Unknown	71 (11.7)	12 (16.4)	7 (13.0)	8 (11.8)	11 (8.1)	25 (16.0)	8 (7.6)	
≤1 cm	181 (29.9)	21 (28.8)	15 (27.8)	23 (33.8)	36 (26.5)	46 (29.5)	37 (34.9)	
>1 cm	353 (58.4)	40 (54.8)	32 (59.3)	37 (54.4)	89 (65.4)	85 (54.5)	61 (57.6)	
Treatment, n (%)								0.20
Surgery	540 (89.3)	64 (87.7)	46 (85.2)	57 (83.8)	123 (90.4)	146 (93.6)	92 (86.8)	0.07
Thyroid hormone	260 (43.0)	24 (32.9)	28 (51.9)	31 (45.6)	70 (51.5)	53 (34.0)	49 (46.2)	0.02
Radiation	320 (52.9)	27 (37.0)	36 (66.7)	29 (42.7)	89 (65.4)	76 (48.7)	57 (53.8)	0.003
Pathology, n (%)								0.13
Papillary ^c^	499 (82.5)	49 (67.1)	43 (79.6)	55 (80.9)	123 (90.4)	133 (85.3)	85 (80.2)	
Follicular	67 (11.1)	16 (21.9)	7 (13.0)	10 (14.7)	8 (5.9)	13 (8.3)	12 (11.3)	
Medullary	6 (1.0)	1 (1.4)	0 (0)	1 (1.5)	0 (0)	3 (1.9)	1 (0.9)	
Anaplastic	17 (2.8)	3 (4.1)	1 (1.9)	0 (0.0)	3 (2.2)	4 (2.6)	6 (5.7)	
Other	16 (2.6)	4 (5.5)	3 (5.7)	2 (2.9)	2 (1.5)	3 (1.9)	2 (1.9)	

^a^ Other racial and ethnic group not presented in table due to low numbers, they were included in the total; ^b^ χ^2^ test for independence for categorical variables and ANOVA for continuous variables comparing racial and ethnic groups; ^c^ papillary and mixed papillary follicular.

**Table 2:** Relative risk of death by sex and race and ethnicity and 5- and 10-year survival among thyroid cancer cases in the Multiethnic Cohort study 1993–2017.

	Men	Women	African American	Filipino	Native Hawaiian	Japanese American	Latino	White	*p* ^d^
Minimally adjusted ^a^									0.08
5-year survival, %	68.7	84.4	73.3	75.9	72.1	83.7	81.1	82.7	
10-year survival, %	52.0	73.2	58.5	61.9	57.0	72.7	68.9	71.2	
HR (95% CI)	2.28 (1.72, 3.01) *	Ref	1.83 (1.18, 2.83) *	1.81 (1.08, 3.04) *	1.95 (1.20, 3.17) *	Ref	1.29 (0.87, 1.91)	1.21 (0.78, 1.88)	
Median survival time	10.2	17.4	12.1	12.9	11.8	17.4	15.2	16.2	
Intermediate ^b^									0.37
5-year survival, %	68.7	85.0	74.7	76.5	73.4	85.0	82.0	83.0	
10-year survival, %	52.0	74.0	59.6	62.3	57.8	74.2	69.8	71.0	
HR (95% CI)	2.04 (1.53, 2.71) *	Ref	1.90 (1.19, 3.04) *	1.56 (0.91, 2.67)	2.46 (1.48, 4.11) *	Ref	1.25 (0.83, 1.88)	1.44 (0.92, 2.27)	
Median survival time	10.8	13.0	13.6	11.3	12.5	10.9	10.9	11.6	
Fully adjusted ^c^									0.04
5-year survival, %	69.5	84.7	78.2	76.8	78.5	82.0	80.8	82.9	
10-year survival, %	52.3	72.9	63.8	62.5	64.2	69.6	67.7	70.6	
HR (95% CI)	2.27 (1.64, 3.15) *	Ref	1.53 (0.91, 2.59)	1.24 (0.70, 2.20)	1.77 (1.01, 3.10) *	Ref	0.98 (0.62, 1.56)	1.12 (0.69, 1.83)	
Median survival time	8.7	17.5	11.3	13.0	11.6	11.9	10.8	10.9	

* Statistically significant. ^a^ Derived from a Cox regression model with the age at diagnosis, race and ethnicity, and sex. ^b^ Additionally adjusted for stage (localized, regional, and distant) as a strata variable. ^c^ Additionally adjusted for treatment, BMI (≤25 kg/m^2^, 25–29.9 kg/m^2^, ≥30 kg/m^2^), smoking status (former, never, current), alcohol intake (grams/day), and nSES. ^d^ *p* value for the global Wald test for race and ethnicity.

**Table 3:** Risk of death comparing thyroid cancer cases compared to matched controls.

	TC Cases	HR (95% CI) ^a^	HR (95% CI) ^b^	HR (95% CI) ^c^
All	605	1.04 (0.91, 1.19)	1.01 (0.87, 1.17)	1.01 (0.87, 1.17)
Sex				
Men	168	1.35 (1.08, 1.69) *	1.39 (1.11, 1.73) *	1.39 (1.11, 1.74) *
Women	437	0.92 (0.78, 1.09)	0.94 (0.79, 1.11)	0.93 (0.79, 1.10)
Race and ethnicity				
African American	73	0.94 (0.67, 1.31)	0.94 (0.67, 1.32)	0.95 (0.68, 1.33)
Filipino	54	1.59 (1.02, 2.48) *	1.62 (1.04, 2.53) *	1.62 (1.04, 2.53) *
Native Hawaiian	68	0.80 (0.53, 1.20)	0.80 (0.55, 1.20)	0.79 (0.52, 1.18)
Japanese American	136	1.25 (0.93, 1.69)	1.29 (0.96, 1.74)	1.29 (0.95, 1.74)
Latino	156	1.09 (0.83, 1.42)	1.10 (0.84, 1.44)	1.10 (0.84, 1.43)
Other	12	0.82 (0.33, 1.99)	0.77 (0.31, 1.88)	0.77 (0.31, 1.88)
White	106	0.93 (0.66, 1.32)	0.97 (0.69, 1.38)	0.98 (0.69, 1.39)

* Statistically significant. ^a^ Cox regression with matched set (age, sex, race and ethnicity) as strata. ^b^ Adjusted by Cox regression with matched set (age, sex, race and ethnicity) as strata and as covariates for BMI (≤25 kg/m^2^, 25–29.9 kg/m^2^, ≥30 kg/m^2^), smoking status (former, never, current), and alcohol intake (grams/day). ^c^ Adjusted by Cox regression with matched set (age, sex, race and ethnicity) as strata and as covariates for BMI (≤25 kg/m^2^, 25–29.9 kg/m^2^, ≥30 kg/m^2^), smoking status (former, never, current), alcohol intake (grams/day), and nSES.

## Data Availability

The data and statistical analysis codes used in this manuscript may be available upon request pending application and approval.

## Supplementary Materials

The following supporting information can be downloaded at: https://www.mdpi.com/article/10.3390/ijerph21030324/s1, Table S1: Relative risk of death by sex and race and ethnicity and 5 and 10-year survival by stage among thyroid cancer cases, the Multiethnic Cohort study 1993–2017.
