# Supplementary material for: Thyroid Cancer Survival in the Multiethnic Cohort Study
Source: Int J Environ Res Public Health. 2024 Mar 10;21(3):324. doi: 10.3390/ijerph21030324 (PMC10970369; doi:10.3390/ijerph21030324)
Supplement: Supplementary file 1 [file ijerph-21-00324-s001.zip › ijerph-2894765-supplementary.pdf]

Table S1. Relative risk of death by sex and race and ethnicity and 5 and 10-year survival by stage among thyroid cancer cases, the Multiethnic Cohort Study 1993-2017.

	**Men**	**Women**	**African American**	**Filipino**	**Native Hawaiian**	**Japanese American**	**Latino**	**White**	***p*** [c]
**Localized**									
Minimally adjusted [a]									
5-year, %	81.3	90.9	83.7	85.2	83.6	89.9	90.3	93.1	
10-year, %	66.5	82.0	70.2	72.6	70.1	80.3	81.1	86.0	
HR (95% CI)	1.85 (1.17, 2.93)*	Ref	1,69 (0.88, 3.25)	1.91 (0.81, 4.52)	1.71 (0.84, 3.48)	Ref	1.00 (0.53, 5.04)	0.83 (0.41, 1.70)	0.27
Fully adjusted [b]									
5-year, %	80.0	91.3	85.6	85.2	85.2	87.6	91.4	92.6	
10-year, %	63.7	82.1	72.4	71.7	71.7	75.7	82.2	84.3	
HR (95% CI)	2.21 (1.53, 3,18)*	Ref	1.12 (0.53, 2.38)	1.44 (0.58, 3.58)	1.20 (0.56, 2.57)	Ref	0.62 (0.31, 1.25)	0.61 (0.28, 1.32)	0.13
**Regional/distant**									
Minimally adjusted [a]									
5-year, %	55.0	73.8	57.2	66.5	50.8	77.2	68.7	60.4	
10-year, %	36.4	58.2	39.2	49.5	32.8	63.0	52.1	42.6	
HR (95% CI)	2.39 (1.36, 4.21)*	Ref	2.70 (1.45, 5.04)*	1.75 (0.90, 3.40)	3.15 (1.55, 6.40)*	Ref	1.63 (0.99, 2.69)	2.39 (1.36, 4.21)*	0.61
Fully adjusted [b]									
5-year, %	54.6	73.6	61.3	66.0	59.2	75.3	69.4	57.9	
10-year, %	36.6	58.3	43.5	48.8	41.2	60.2	52.7	40.6	
HR (95% CI)	1.85 (1.17, 2.93)*	Ref	1.53 (0.72, 3.25)	1.55 (0.77,3.13)	1.66 (0.74,3.77)	Ref	1.47 (0.82, 2.64)	1.95 (1.02,3.72)*	0.68

*Statistically significant

[a] Derived from a Cox regression model with age at diagnosis, race and ethnicity, and sex

[b] Additionally adjusted for treatment, BMI (≤25, 25-29.9, ≥30 kg/m^2), smoking status (former, never, current), alcohol intake (g/day) and nSES.

[c] p value for global Wald test for race and ethnicity.
